# Factors associated with early mycological clearance in HIV-associated cryptococcal meningitis

**DOI:** 10.1371/journal.pone.0174459

**Published:** 2017-03-29

**Authors:** Fátima Concha-Velasco, Elsa González-Lagos, Carlos Seas, Beatriz Bustamante

**Affiliations:** 1 Instituto de Medicina Tropical “Alexander von Humboldt”, Universidad Peruana Cayetano Heredia, Lima, Perú; 2 Facultad de Medicina, Universidad Peruana Cayetano Heredia, Lima, Perú; 3 Departamento de Enfermedades Infecciosas, Tropicales y Dermatológicas, Hospital Cayetano Heredia, Lima, Perú; University of Minnesota, UNITED STATES

## Abstract

**Introduction:**

The first-line combination therapy for HIV-associated cryptococcal meningitis (CM), a condition of high mortality particularly in the first two weeks of treatment, consists of amphotericin B plus flucytosine (5-FC). Given that 5-FC remains unavailable in many countries, the knowledge of factors influencing mycological clearance in patients treated with second-line therapy could contribute to effective management.

**Objectives:**

To determine the factors associated with the clearance of *Cryptococcus* sp. from the cerebrospinal fluid by the second week of effective antifungal therapy (early mycological clearance) in HIV-associated CM.

**Methods:**

Retrospective cohort study based on secondary data corresponding to HIV-associated CM cases hospitalized at a tertiary health care center in Lima, Peru where 5-FC remains unavailable. Risk factors associated with early mycological clearance were analyzed by generalized linear regression models.

**Results:**

From January 2000 to December 2013, 234 individuals were discharged with a diagnosis of HIV-associated CM; in 215 we retrieved the required data. The inpatient mortality was 20% (43/215), 15 of them in the first two weeks of treatment. In the final model (157 cases), adjusted for age, previous episode of CM, ART use, type of antifungal treatment, raised intracranial pressure, frequency of therapeutic lumbar punctures, baseline fungal burden and treatment period, the factors associated with early mycological clearance were: Amphotericin B deoxycholate plus fluconazole as combination therapy (RR, 1.56; 95% CI, 1.14–2.14); severe baseline intracranial pressure (≥35 cm H_2_O) (RR, 0.57; 95% CI, 0.33–0.99); and baseline fungal burden over 4.5 log_10_ CFU/mL (RR, 0.61 95% CI: 0.39–0.95).

**Conclusions:**

In a setting without access to first-line therapy for CM, the combination therapy with amphotericin B deoxycholate plus fluconazole was positively associated with early mycological clearance, while high fungal burden and severe baseline intracranial pressure were negatively associated, and thus related to failure.

## Introduction

Notwithstanding antiretroviral therapy (ART) has reduced the global burden of cryptococcal meningitis (CM), CM still affects approximately one million people per year [1,2]. Indeed, CM remains an important cause of disability and mortality particularly for people living with HIV in Africa, Asia and South America [1].

Early mortality rates of CM, defined by deaths that occur within the first two weeks of treatment, vary from 5.6% in developed countries [3] to 10–25% in Africa [4]. Given that flucytosine (5-FC) is generally unavailable in Africa, Asia and South America [5], the difference in mortality probably responds to hampered access to the recommended first-line therapy for CM, which includes amphotericin B (AmB) and 5-FC for, at least, the initial two-week induction period [6]. Wherever 5-FC remains unavailable, therapy for CM alternatively considers AmB and/or fluconazole; yet, additional limitations may preclude the safe use of AmB and potentially derive into fluconazole monotherapy, which is associated with increased CM mortality [7–9].

Early mortality of CM is also associated with high fungal burden [10, 11]. Fungal burden, altered mental status and rate of fungal clearance were the predictors for early mortality in a large cohort study conducted in Africa; in that study, there were different schemes of CM therapy, including fluconazole monotherapy in a small group [12]. In another study in Peru, early mortality of CM was related with Glasgow coma scores <14 and CSF cryptococcal antigen titers >1:1024; however, not all CM cases were confirmed by isolation of *Cryptococcus* in culture [13].

Despite the extensive use of AmB plus fluconazole as combination therapy for CM in resource-limited settings, there is limited evidence about its fungicidal activity and the early mortality among patients who receive such alternative treatment. Compared to AmB plus fluconazole and to AmB monotherapy, AmB plus 5-FC is superior for cryptococcal clearance and patient survival [14,15]. The early fungicidal activity of fluconazole was superior at 1200 mg/d vs 800 mg, but the mortality was similar between both groups, at two and ten weeks of treatment [16]. In a small study in South Africa, the early fungicidal activity observed in patients treated with either fluconazole or voriconazole combined with AmB was not statistically different from that observed in patients treated with AmB plus 5-FC [17].

In this study, conducted in a setting where 5-FC remains unavailable, we evaluated the factors associated with early mycological clearance in HIV-associated CM.

## Material and methods

### Study design and population

We retrospectively studied all cases of HIV-associated CM admitted at our study center between January 2000 and December 2013. The study center, a Tertiary Health Care Facility in Lima, has a referral unit for infectious and tropical diseases; this unit has the largest number of HIV patients under care in Peru.

**Fig 1:**
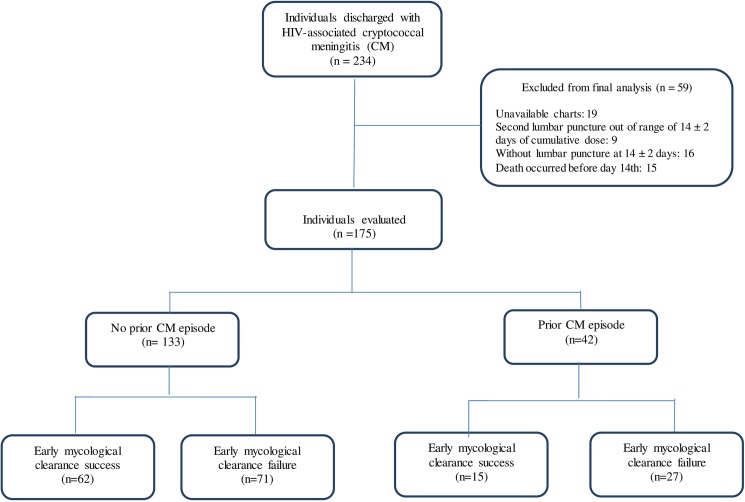
Flowchart of selection of study population among cases discharged with diagnosis of HIV-associated cryptococcal meningitis (2000–2013).

We included the cases that met the following criteria: confirmed HIV-infection, age ≥18 years at the time of admission for CM, and at least one episode of CM confirmed by isolation of *Cryptococcus* sp. from CSF culture. For the main analysis, the exclusion criterion was the lack of a lumbar puncture at the end of the induction phase (Fig 1).

### Data sources, data collection and laboratory tests

We considered four data sources: the database of discharge diagnoses; the clinical records; the reports from the mycology laboratory at IMTAvH; and the database from the HIV program. In order to identify the eligible population, we first searched and retrieved all diagnosis of CM, stored in text and ICD-10 codes. Then, we reviewed the clinical records and filled out a study form of clinical and laboratory data of interest such as HIV status, previous episodes of CM, antifungal therapy for the current CM episode, antiretroviral therapy at the time of CM diagnosis, and fungal cultures at baseline and during follow-up.

The results of fungal cultures and time to growth were cross-checked with the original reports at the mycology laboratory. The data on CD4 values, viral load and antiretroviral therapy was retrieved from the HIV program database, considering a timeframe of six months prior and posterior to the date of admission for the CM episode. As part of the routine services of the HIV program, CD4 cell counts (cells/mm^3^) and viral load (log_10_ copies/mL) determinations were performed at a national referral laboratory. Additional laboratory data was retrieved from clinical charts as reported by the routine clinical laboratories at the study center.

During the study period, the microbiological identification of *Cryptococcus* colonies in patients hospitalized at the study center was routinely performed by standard methods in the mycology laboratory at IMTAvH. Since 2005, the personnel of such laboratory has been participating in The College of American Pathologists Proficiency Testing Programme (CAP) to identify yeasts, susceptibility *in-vitro* to antifungals, direct examination with India ink stain and CSF cryptococcal antigen detection test.

### Definitions of main study variables

Consistent with our eligibility criteria, all included cases had *Cryptococcus* sp. in the initial CSF sample. The main outcome variable was early mycological clearance, defined by a negative culture (no *Cryptococcus* sp.) in a CSF sample taken by the second week of effective antifungal therapy. Cryptococcal cultures were considered negative if *Cryptococcus* sp. was not isolated up to five weeks after incubation. Effective antifungal therapy was defined by completion of the induction phase of therapy. The number of days from the incubation of culture to the detection of isolates defined the *Cryptococcus* sp. growth rate; the number of colony forming units (CFU) (log_10_/ml) in the initial culture defined the baseline fungal burden.

### Data management and statistical analysis

Data was entered into an excel data base and exported to Stata version 13 (StataCorp, College Station, Texas). Extensive data cleaning and quality control processes were undertaken to assure data integrity prior to final statistical analyses.

We compared baseline characteristics between included and excluded subjects. In the case of subjects with more than one CM episode, only the last episode was considered in the final analysis. We conducted descriptive and bivariate analysis; categorical variables were compared using chi-square or Fisher´s exact tests. For the main analysis, factors independently associated with early mycological clearance, robust generalized Poisson regression linear models were performed in bivariate and multivariate analysis considering early mycological clearance as the outcome variable. Results are presented as risk ratios (RR) and 95% confidence intervals (ICs). The final multivariate model was conducted through backward-forward stepwise technique; no imputation was performed for missing variables.

The factors considered in the model were age (18–39 and ≥ 40 years); previous episode of CM; ART use; antifungal treatment type ([AmB deoxycholate 0.6 mg/Kg/d plus fluconazole 800 mg] or [AmB monotherapy 0.6 mg/Kg/d]); complementary use of interferon; raised intracranial pressure (IP) (mild [20–24 cmH_2_O], moderate [25-34cmH_2_O] and severe [≥ 35cmH_2_O]); frequency of therapeutic lumbar punctures (<4, ≥4); baseline fungal burden (lower third: 0.2 to 3.2, middle third: 3.3 to 4.5 and upper third: 4.5 to 6.1 CFU log_10_/mL) and treatment period, according to the ART provision by the government of Peru (2000–2005, pre ART provision and ≥ 2006–2013 post ART provision of ART).

### Ethical considerations

Data collection and analysis preserved the confidentiality of information. We used codification in the management of the databases to safeguard the identity of the individuals from the study. Data access was restricted to authors. The study protocol was reviewed and approved by two independent Ethics Committees: UPCH Institutional Ethics Committee Review Board (reference 62181) and HNCH Institutional Ethics Committee Review Board (reference 108–013). UPCH Institutional Ethics Committee is registered in the Office for Human Research Protections-OHRP: IORG0000671 and IRB00001014 and Federal Wide Assurance: FWA00000525.

## Results

### Study population and clinical outcomes

During the study period, 234 individuals were discharged with a diagnosis of HIV-associated CM. The information required for this study was available in 215 individuals (91.9%), as in 19 cases the clinical charts or reports from the mycology laboratory were missing (Fig 1). The overall inpatient mortality was 20% (43/215); 15 out of 215 (7.0%) died before two weeks of treatment. Overall, there were no differences on baseline fungal load between CM cases discharged alive (3.39 ± 1.28 log_10_ CFU/mL) and those deceased (3.67 ± 1.41 log_10_ CFU/mL). However, after the second week of treatment, there were differences on baseline fungal load between cases who died and those who were discharged alive (4.21 ± 0.21 log_10_ CFU/mL vs. 3.39 ± 0.10 log_10_ CFU/mL, p = 0.002).

For the final analysis of early mycological clearance, we considered 175 cases (74.8%), given that 25 did not have a lumbar puncture at 14 ± 2 days; 15 (7.0%) of them died in the first two weeks after admission. A prior episode of CM was registered in 24% (n = 42/175); among 30 with information on exact dates, the median time between the CM episodes was 181 days (RIQ: 142–335) days.

In our setting, and since the beginning of the study period, therapeutic lumbar punctures were frequently used as part of the routine care. During the first two weeks of therapy, one third of the cases in this study had more than four lumbar punctures, although data on CSF drainage volume was missing.

The comparison of baseline characteristics between the cases that were included and excluded in the final analysis showed similarities in terms of age, history of CM, CD4 T-cell count, HIV viral load, ART use and treatment period. The mortality was lower in those included (14.3%, n = 25) than in those excluded, which could be explained by the inclusion criteria of an available lumbar puncture at 14 ± 2 days. The mortality was higher in the group without history of CM (15.8%) than in those with previous CM (7.1%). During the induction phase, deaths occurred in 9% (n = 13) of those who received AmB monotherapy and in 6.3% (n = 3) of those who received AmB in combination with fluconazole. There were additional differences in antifungal treatment, frequency of headache, meningeal signs and fungal burden (Table 1).

**Table 1:** Comparison of baseline clinical and laboratory characteristics between included and excluded cases with HIV-associated CM in Peru*.

		N = 215	Included (n = 175)	Excluded (n = 40)	p
Male, n (%)		215	134 (76.6)	28 (70.0)	0.418
Age–years		215	34.8 ± 9.0	33.7 ± 11.8	0.573
Previous episode of CM^†^, n (%)		215	42 (24.0)	11 (27.2)	0.215
Duration of symptoms, median (IQR)–days		207	18/167 (10–30)	20/40 (12.5–60)	0.82
CD4 cell count, median (IQR)—cel/mm^3^		161	33/138 (14–81)	26/23 (20–50)	0.367
HIV load–copies log_10_/mL^‡^		130	4.6/113 ± 1.4	4.6/17 ± 1.3	0.573
Antiretroviral use, n (%)		31	24 (13.7)	7 (17.5)	0.539
Inpatient mortality, n (%)		215	25 (14.3)	18 (45.0)	<0.001
Antifungal treatment, n (%)		215			0.001
Amphotericin B		145	109 (62.3)	36 (90.0)	
	Amphotericin B plus fluconazol	48	44 (25.1)	4(10.0)	
	Amphotericin B plus interferon	22	22(12.6)	0 (0.0)	
Treatment period, n (%)—years		215			0.183
	2000–2005	114	89 (50.9)	25 (62.5)	
	≥2006–2013	101	86 (49.1)	15 (37.5)	
Clinical parameters, n (%)					
	Headache	179	151 (86.3)	28 (70.0)	0.013
	Vomiting	123	103 (58.9)	20 (50.0)	0.307
	Fever	120/213	100/173 (57.8)	20/40 (50.0)	0.37
	Seizures	24	20 (11.4)	4 (10.0)	0.796
	Signs of neurological focalization	13	11 (6.3)	2 (5.0)	0.758
	Altered mental status	42	37 (21.1)	5 (12.5)	0.214
	Meningeal signs	95/213	87/173 (50.3)	8/40 (20.0)	<0.001
	Glasgow scale score	189	14.6/163 ± 0.10	14.8/26 ± 0.5	0.241
	Baseline CSF opening pressure—cmH_2_O^§^	197	29.6/165 ± 13.2	26.3/32 ± 13.5	0.2
	Frequency of serial lumbar punctures, median (RI)	193	3/162 (2–4)	3/31 (2–4)	0.342
Baseline laboratory parameters, n (%)					
	QCC—log_10_CFU/mL ^¶^	195	3.5/163 ± 1.2	2.6/32 ± 1.4	0.001
	*Cryptococcus* growth rate, median (IQR)–days	165	4 (2–5)	4 (3–6)	0.73
	Cryptococcal antigen testing, median (IQR)	178	2048/148 (256–8192)	1536/30 (32–4096)	0.796
	CSF white cell count, median (IQR)—cell/uL	182	3/151 (0–14)	0/31 (0–4)	0.03
	CSF glucose level—mg/dL	180	35.6/153 ± 14.3	34.9/27 ± 16.4	0.81
	CSF proteins level, median (IQR)—g/L	180	70/152 (42.5–106)	87.5/28 (62.5–120)	0.217

* Values represent the mean± standard deviation unless otherwise specified.

^**†**^ CM: cryptococcal meningitis.

^‡^ HIV load: number of HIV virus in blood.

^§^ CSF: cerebro spinal fluid.

^¶^ QCC: quantitative cryptococcal culture.

### Mycological characteristics in the population included in the final analysis

The baseline median fungal burden was 3.5 ± 1.2 log_10_ CFU/mL; among 136 cases with information for *Cryptococcus* sp. growth rate, the median was 4 days (IQR: 2–5). Most of the baseline strains (85.2%; 116/136) grew in less than 7 days and only 0.7% did it after 14 days. The median baseline fungal burden, as well as the baseline *Cryptococcus* sp. growth rate, were similar regardless previous episodes of CM: 3.5 ± 1.2 log_10_ CFU/mL in those with history of CM and 3.4 ± 1.2 log_10_ CFU/mL in those without; 4 (IQR: 2–5) days in those with history of CM, and 3.5 (IQR: 2–6) days in those without.

Slight positive linear correlation was found between baseline fungal burden and CSF cryptococcal antigen titer (Pearson = 0.21; p = 0.014), and between baseline fungal burden and baseline CSF opening pressure (Pearson = 0.28; p = 0.001) (S1 Fig, S2 Fig). A slight inverse correlation was observed between baseline fungal burden and *Cryptococcus* sp. growth rate (Pearson = -0.27, p = 0.002) (S3 Fig).

### Early mycological clearance: Frequency and risk factors

Early mycological clearance was observed in 77 (44%) of the 175 CM cases included in the final analysis. In bivariate analyses, early mycological clearance was associated with lower baseline fungal burden and lower baseline CSF opening pressure. Indeed, fungal burden over 4.5 log_10_ CFU/mL and severe baseline IP (≥35 cm H_2_O) were associated with early mycological clearance failure (Table 2).

**Table 2:** Comparison of baseline characteristics according to the status of early mycological clearance (success vs failure) among cases with HIV-associated CM in Peru*.

			Early mycological clearance		
Characteristics		N = 175	Success (n = 77)	Failure (n = 98)	p
Age—years		175	33.5 ± 7.9	35.9 ± 9.7	0.076
CD4 load, n (%)		137			
	<100	108	52 (78.8)	56 (78.9)	1.000
	100–199	20	10 (15.2)	10 (14.1)	
	200–499	9	4 (6.1)	5 (7.0)	
HIV load—copies log_10_/mL^‡^^Δ^		113	4.5/54 ± 1.5	4.8/59 ± 1.3	0.190
Baseline CSF opening pressure—cmH_2_O^§^^Δ^		165	27.3/74 ± 14.4	31.6/91 ± 12	0.040
Antiretroviral use, n (%)		18/175	11 (14.3)	7 (7.1)	0.123
Previous episode of CM^†^, n (%)		42/175	15 (19.5)	27 (27.6)	0.250
Antifungal treatment, n (%)		175			0.050
	Amphotericin B	109	44 (57.1)	65 (66.3)	
	Amphotericin B plus fluconazol	44	26 (33.8)	18 (18.4)	
	Amphotericin B plus interferon	22	7 (9.1)	15 (15.3)	
Frequency of serial lumbar punctures, n (%)		170			0.047
	<4	92	47 (62.7)	45 (47.4)	
	≥4	78	28 (37.3)	50 (52.6)	
QCC—log_10_ CFU/mL^¶^^Δ^		163	3.3/74 ± 1.2	3.9/89 ± 0.1	0.001
*Cryptococcus* growth rate, median (IQR)—days^Δ^		136	4/56 (2–6.5)	3.5/80 (3–5)	0.089
Treatment period, n(%)—years		175			0.029
	2000–2005	89	32 (41.2)	57 (58.2)	
	≥ 2006–2013	86	45 (58.4)	41 (41.8)	

* Values represent the mean ± standard deviation unless otherwise specified.

Δ The values are from the number of samples tested.

^**†**^ CM: cryptococcal meningitis.

^‡^ HIV load: number of HIV virus in blood.

^§^ CSF: cerebro spinal fluid.

^¶^ QCC: quantitative cryptococcal culture.

In the final model, adjusted by baseline CSF opening pressure, fungal burden, age, type of antifungal treatment, previous episode of CM, treatment period and frequency of serial lumbar punctures, there were three factors independently associated with early mycological clearance: severe baseline IP (≥35 cm H_2_O) (RR, 0.57; 95% CI, 0.33–0.99); combination of antifungal therapy (RR, 1.56; 95% CI, 1.14–2.14); and baseline fungal burden over 4.5 log_10_ CFU/mL (RR, 0.61 95% CI: 0.39–0.95). The results of the Poisson regression analysis model for early mycological clearance are displayed in Table 3.

**Table 3:** Risk factors associated with early mycological clearance in cases with HIV-associated CM in Peru*.

		Bivariate analysis		Multivariate analysis	
Characteristics		RR (95% CI)	p	RR (95% CI)	p
Age–years					
	≥ 40	Reference			
	18–39	1.40 (0.84–2.33)	0.198		
Previous episode of CM†, n (%)					
	No	Reference			
	Yes	0.93 (0.58–1.51)	0.775		
Antiretroviral use, n (%)					
	No	Reference			
	Yes	1.43 (0.93–2.21)	0.104		
Antifungal treatment, n (%)					
	Amphotericin B	Reference		Reference	
	Amphotericin B plus fluconazol	1.40 (1.00–1.98)	0.052	1.56 (1.14–2.14)	0.005
	Amphotericin B plus interferon	0.73 (0.38–1.40)	0.340	0.85 (0.42–1.69)	0.636
Baseline CSF opening pressure—cmH_2_O^§^					
	Absent (< 20)	Reference		Reference	
	Mild (20–24)	0.92 (0.57–1.49)	0.743	0.85 (0.54–1.32)	0.463
	Moderate (25–34)	0.99 (0.67–1.46)	0.962	0.80 (0.53–1.20)	0.276
	Severe (≥ 35)	0.50 (0.28–0.90)	0.021	0.57 (0.33–0.99)	0.046
Frequency of serial lumbar punctures, n (%)					
	< 4	Reference			
	≥ 4	0.75 (0.52–1.08)	0.119		
QCC—log10 CFU/mL^¶^					
	Lower third (0.18–3.23)	Reference		Reference	
	Middle third (3.24–4.47)	0.92 (0.64–1.33)	0.661	0.92 (0.65–1.30)	0.621
	Upper third (4.48–6.11)	0.55 (0.35–0.89)	0.014	0.61 (0.39–0.95)	0.028
Treatment period, n (%)–years					
	2000–2005	Reference			
	≥ 2006–2013	1.34 (0.94–1.91)	0.104		

*Model adjusted for baseline CSF opening pressure, fungal burden, age, type of antifungal treatment, previous episode of CM, treatment period and frequency of serial lumbar punctures.

†CM: cryptococcal meningitis.

§ CSF: cerebro spinal fluid.

¶ QCC: quantitative cryptococcal culture.

## Discussion and conclusions

In a setting without access to the first-line therapy for HIV-associated CM, we found that early mycological clearance was associated with the use, during the induction phase, of AmB deoxycholate plus fluconazole as combination therapy, in comparison to AmB monotherapy; with severe baseline CSF opening pressure and with high fungal burden at entry: a value over 4.5 log_10_ CFU/mL was indeed associated with failure.

For HIV-associated CM, AmB plus 5-FC therapy leads to the best results in terms of early fungal clearance and mortality in a 10-week period [15]. Our study was performed in a country where, as in many others worldwide, 5-FC remains unavailable; therefore, the regimens in use were AmB deoxycholate plus fluconazole and even AmB alone. In this setting, early mycological clearance was favored with AmB deoxycholate plus fluconazole combination therapy. Although AmB and fluconazole are theoretically antagonistic drugs, *in vitro* and clinical observations support the effectiveness of the combination [10,18,19].

Fluconazole, a well-tolerated and widely available azole with significant *in vitro* activity against most strains of *Cryptococcus neoformans*, is recommended for patients with CM who have responded to the induction therapy with AmB [6]. In a study in Vietnam, the mortality appeared to be lower in the group of combination therapy with AmB deoxycholate plus fluconazole than in the group with AmB monotherapy, both at two weeks (25.3% vs 20.2%) and at ten weeks (33.3% vs 44.4%), though these differences were not significant [15]. However, in a recent meta-analysis that remarked the benefits of AmB in combination with 5-FC, over AmB monotherapy, for patients with altered levels of consciousness, no major advantages were reported for the combination of AmB with azoles [20]. Clearly, there is need for additional evidence that compares the effectiveness of the combination therapy of amphotericin B plus fluconazole vs AmB monotherapy. This will allow to definitely establishing if, as suggested by our findings, such combination therapy has indeed a superior effect that fastens clearance of the infection and reduces early CM mortality in settings where 5-FC is not available.

High fungal burden at baseline is related to mortality in CM [10, 12]. A negative CSF culture at the end of the induction phase is an important predictor of fungal clearance at 10 weeks [21]. There is a positive correlation between fungal burden and increased IP [22]: the accumulation of fungi at the arachnoid villi and subarachnoid spaces generates an inflammatory reaction that obstructs CSF outflow and increases IP. Moreover, high levels of CSF IL-10 are observed among CM patients with high fungal burden, which also leads to decreased levels of CSF TNF-α, IFN-γ, IL2 and IL-17A, ultimately stimulating pathways that raise mortality [23]. Therefore, a high baseline fungal burden (over 4.5 log_10_ CFU/mL) could be related to failure in mycological clearance at 10 weeks in HIV-associated CM. Slower fungicidal activity, determined by the decrease in log_10_ CFU/mL CSF per day, has been proposed as a useful prognostic indicator of high CM mortality, at 2 and 10 weeks of antifungal therapy [11,12,24].

We found that high baseline IP (≥35cmH_2_O) was associated with failure of early mycological clearance. As previously described, high opening pressure could respond to the higher fungal burden in a subgroup of patients, as it was associated with higher baseline CSF cryptococcal antigen titer, with headache, meningismus, papilloedema, hearing loss, and poorer short-term survival [22,25,26]. In fact, high opening pressure demands CSF drainage in large volumes, which support the contributory survival effect of therapeutic lumbar punctures [26].

In a study with 26 weeks of follow-up, survival of HIV-associated CM was higher when ART initiation was deferred for five weeks rather than for two weeks [2]. With regard to fungal clearance in CSF, ART-experienced and ART-naive CM patients had similar results; however, ART-experienced patients had lower baseline fungal burden and longer survival [27]. In that study, the number of ART-experienced patients was small, and 7.1% were not virally suppressed despite history of ART use.

According to our findings, early mycological clearance was not associated with *Cryptococcus* growth rate. Although the recommended incubation period for *Cryptococcus* CSF is four to six weeks [28,29,30], the time required to finally report a CSF culture as negative for *Cryptococcus* is not well defined. In this study, negative results of CSF cultures were reported after five weeks, although most of the positive CSF cultures grew before two weeks.

The main limitations of this study are basically derived from the use of routinely gathered data, and the changes in health care practices across the timespan considered. For instance, AmB deoxycholate plus fluconazole as combination therapy was not in use at our center before 2006; data on CSF drainage volume, scores on Glasgow coma scale, and altered mental status at entry were limited. We did not impute missing values and were thus probably underpowered to analyze the association of *Cryptococcus* sp. growth rate. At the same time, the major strength of this study consists on the use of data collected in a center where the mycology laboratory followed standardized and consistent procedures throughout the whole study period.

Despite these limitations, our information contributes to describe factors of early mycological clearance in HIV-associated CM in a Latin American setting where, in view of the unavailability of 5-FC, second-line treatment of AmB deoxycholate plus fluconazole combination therapy stands as the best alternative. Latin America ranks third in the number of cases with HIV-associated CM, being thus in clear need of evidence-based interventions to optimize the care of these patients; however, the related publications are scarce [31]. Accordingly, we insist that further studies are needed to evaluate the effects of antifungal combination on early mycological clearance and survival. In addition, we should also remark that it is imperative to expand the access to 5-FC by its inclusion in the Essential Medicines List of hospitals.

In conclusion, early mycological clearance in HIV-associated CM is favored by AmB plus fluconazole combination therapy, whereas it is affected by high fungal burden (>4.5 log_10_ CFU/mL) and severe CSF opening pressure (≥35 cmH_2_O) at entry. In clinical settings, identification of these factors merits a thorough follow-up.

## Supporting information

S1 Fig Relationship between baseline fungal burden and CSF cryptococcal antigen titer. (TIF)

S2 Fig Relationship between baseline fungal burden and baseline CSF opening pressure. (TIF)

S3 Fig Relationship between baseline fungal burden and *Cryptococcus* sp. growth rate. (TIF)

S1 File Dataset. (DTA)
