# Supplementary figures and images for: Factors associated with early mycological clearance in HIV-associated cryptococcal meningitis
Source: PLoS One. 2017 Mar 29;12(3):e0174459. doi: 10.1371/journal.pone.0174459 (PMC5371305; doi:10.1371/journal.pone.0174459)

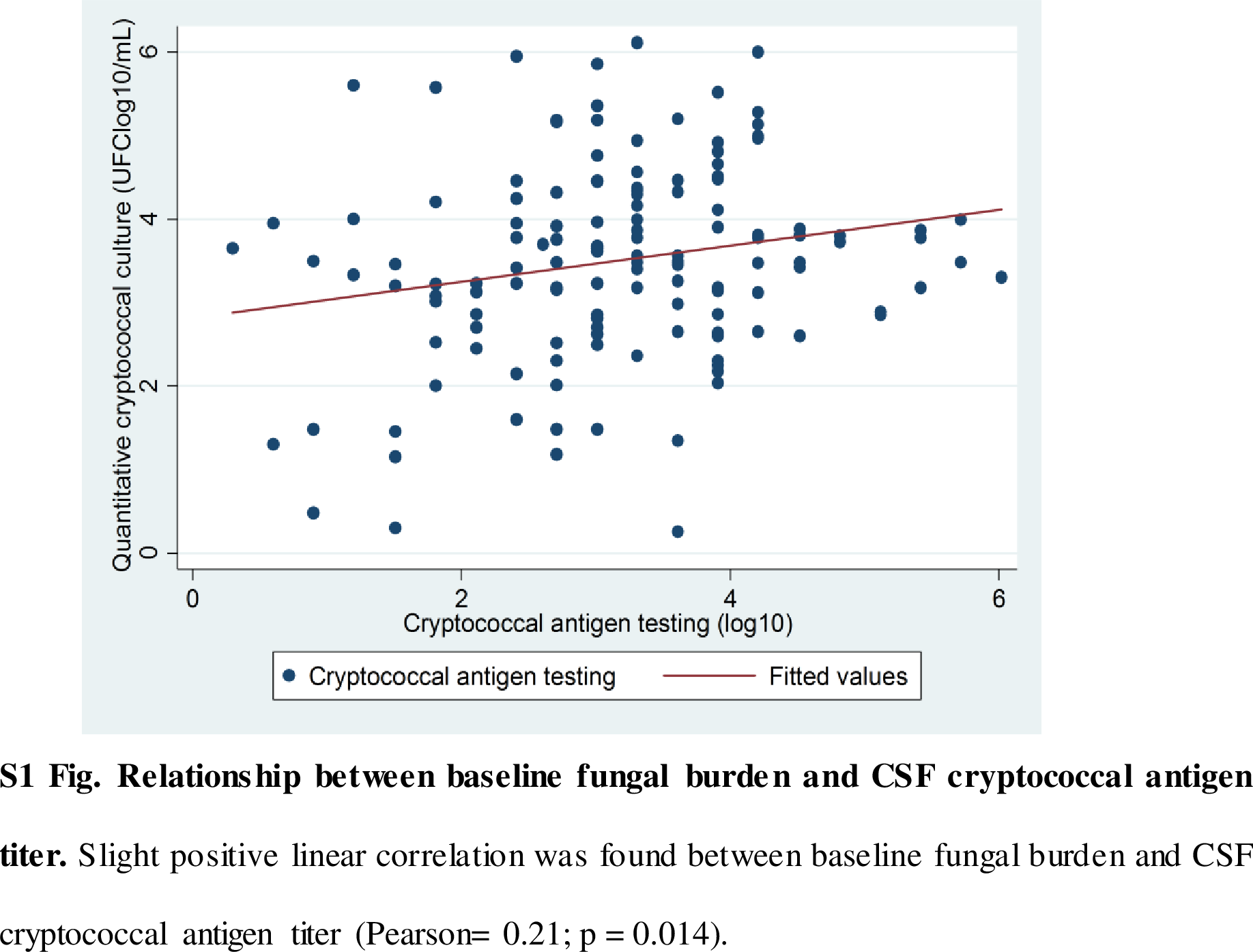

Supplement: S1 Fig — (TIF) [file pone.0174459.s001.tif]

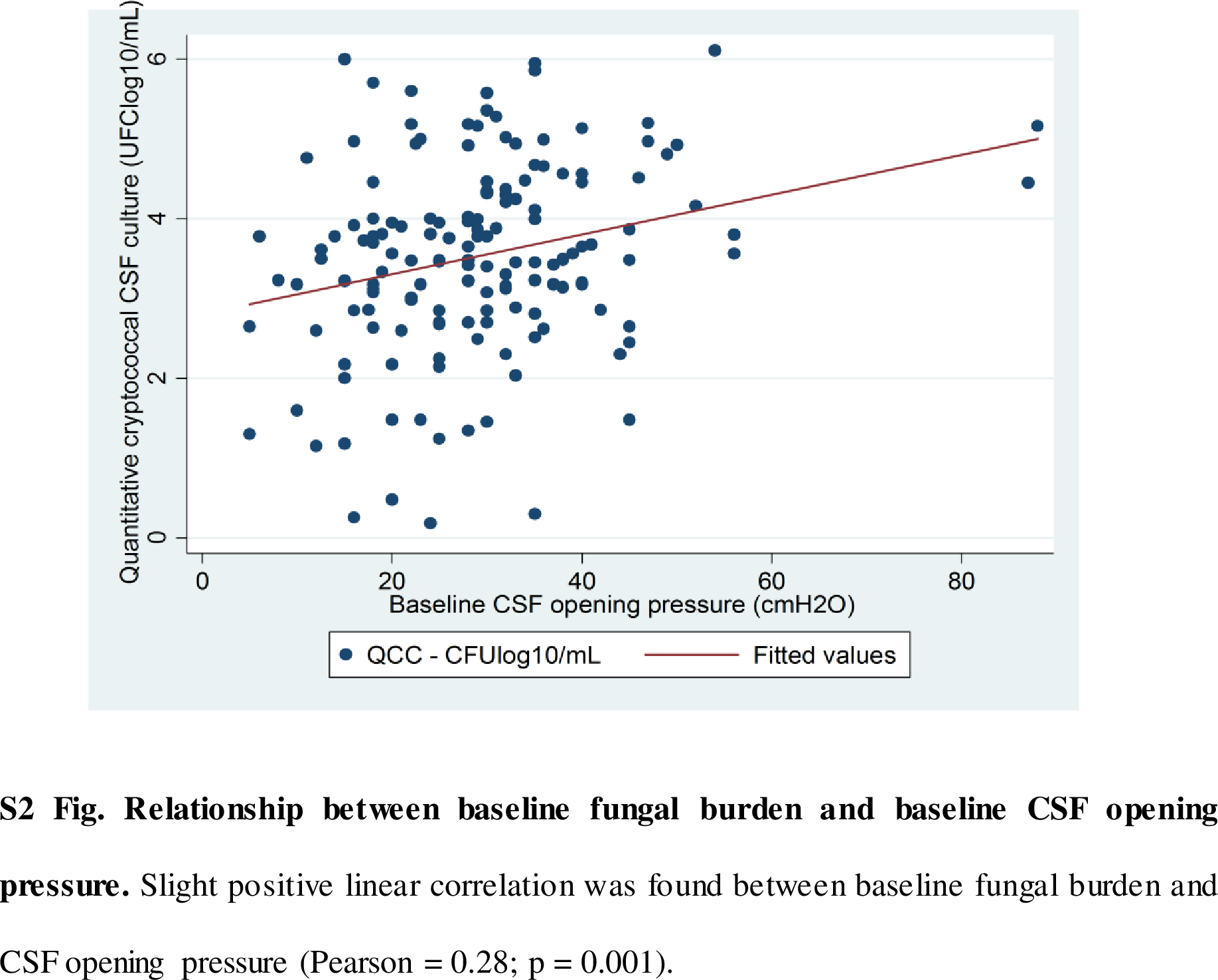

Supplement: S2 Fig — (TIF) [file pone.0174459.s002.tif]

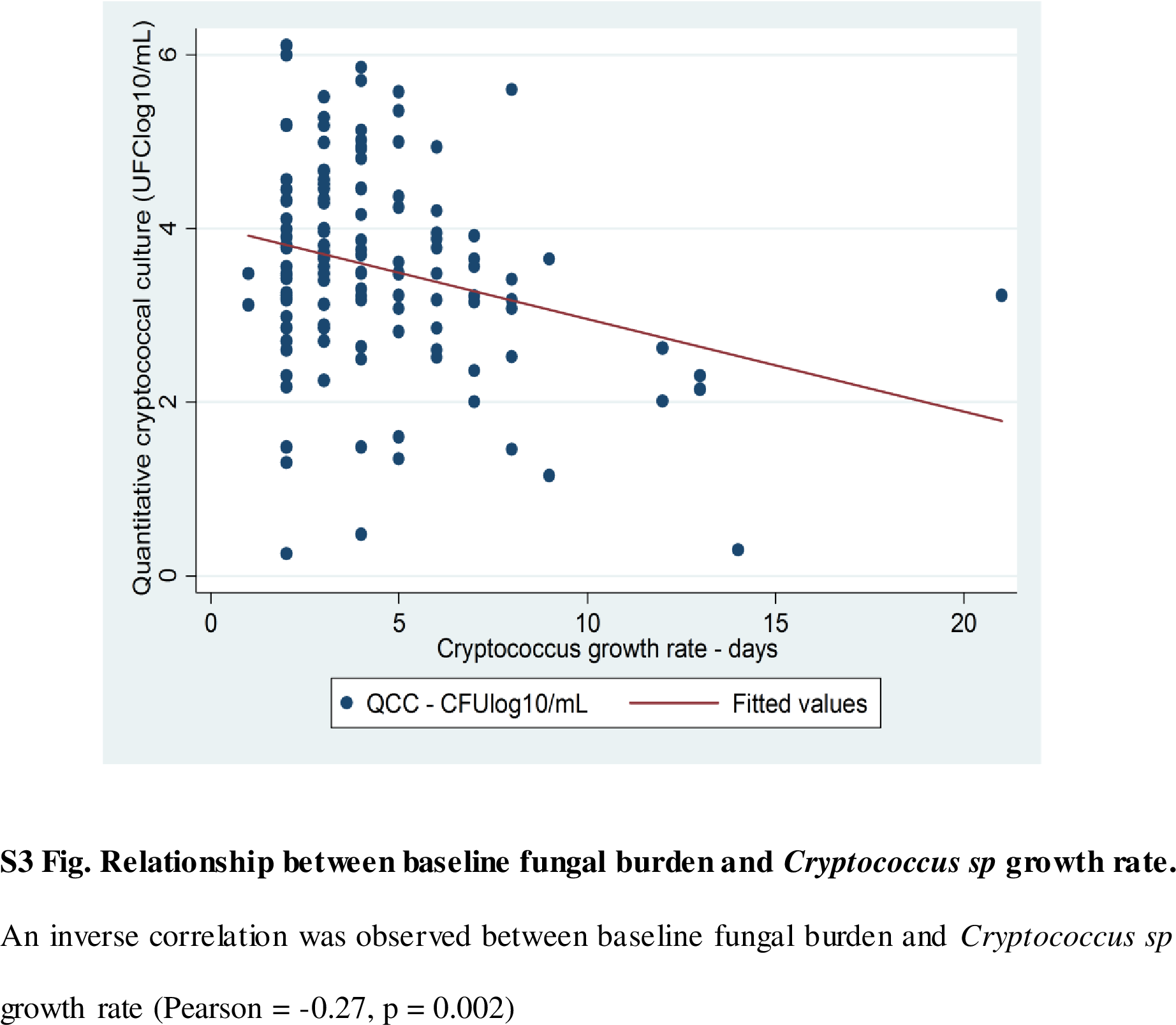

Supplement: S3 Fig — (TIF) [file pone.0174459.s003.tif]
